# Licorice-Induced Pseudohyperaldosteronism Presenting as Seizure with Posterior Reversible Encephalopathy Syndrome: A Case Report

**DOI:** 10.51894/001c.165618

**Published:** 2026-07-31

**Authors:** Al-Noor Ikram, Georges Nakhoul, Katharine D. Currie, Jill M. Slade

**Affiliations:** 1 College of Osteopathic Medicine Michigan State University

## 88

### Introduction

Uncontrolled hypertension can lead to a hypertensive crisis, which can be associated with a multitude of etiologies, including endocrine disorders, sleep apnea, vascular stenosis, or medication and supplement use. This case highlights licorice-induced pseudohyperaldosteronism as a reversible and underrecognized cause of hypertensive emergency and posterior reversible encephalopathy syndrome (PRES), particularly in older adults with complex medical histories.

### Case Description

A 72-year-old woman with a history of hypertension and carotid artery disease presented to the ER with acute severe hypertension recorded at 210/90, associated with posterior headache, new-onset seizures, and altered mental status. She was unresponsive with a Glasgow Coma Scale score of 7 and required emergent intubation. Initial laboratory evaluation revealed hypokalemia, metabolic acidosis, and hyperglycemia. Neuroimaging with CT and CTA showed no acute hemorrhage or aneurysmal rupture. Brain MRI demonstrated symmetric posterior cerebral and cerebellar FLAIR hyperintensities consistent with PRES. The patient was treated with antiepileptic therapy, propofol, electrolyte repletion, and ventilatory support, with rapid neurologic recovery following stabilization. Abdominal MRI showed no adrenal lesions and catheterization ruled out significant renal stenosis. The patient was discharged with aggressive medication to control blood pressure without a clear understanding of the cause of the hypertensive emergency. Outpatient testing ruled out pheochromocytoma and scleroderma. Low serum renin and aldosterone was present along with a history of black licorice candy consumption, leading to a differential diagnosis of licorice-induced pseudohyperaldosteronism.

### Discussion

This case is notable because the precipitating agent for the hypertensive crisis was candy flavored with licorice extract. Licorice extract is FDA approved and widely consumed without specific regulations to prevent toxicity. Products containing licorice root or extract carry an unrecognized risk of inducing severe hypertension. Licorice extract contains glycyrrhizic acid, which inhibits 11β-hydroxysteroid dehydrogenase type 2, allowing cortisol to activate mineralocorticoid receptors. This mechanism produces pseudohyperaldosteronism characterized by hypertension, hypokalemia, and metabolic alkalosis. The patient’s hypertension led to vasogenic edema in the brain manifesting as PRES inducing seizure. By highlighting this uncommon presentation, this report aims to increase awareness among clinicians and encourage a broader differential diagnosis in similar scenarios to aid in decreasing serious health outcomes.

